# The Acidic Fraction of Isatidis Radix Regulates Inflammatory Response in LPS-Stimulated RAW264.7 Macrophages through MAPKs and NF-*κ*B Pathway

**DOI:** 10.1155/2021/8879862

**Published:** 2021-03-09

**Authors:** Zhenyu Fan, Liangliang Cai, Yage Wang, Qiuyan Zhu, Shengnan Wang, Bohua Chen

**Affiliations:** ^1^Department of Pharmacy, The Affiliated Hospital of Nantong University, Nantong 226000, China; ^2^Department of Microsurgery, The Orthopaedic Hospital of Henan Province, Luoyang 471000, China

## Abstract

Isatidis Radix, the dried root of *Isatidis indigotica* Fort, is a traditional heat-clearing and detoxicating herb, which has the antiviral and anti-inflammatory activity and immune regulation. It has been widely used to treat cold, fever, sore throat, mumps, and tonsillitis in clinics. A previous study demonstrated that the acidic fraction of Isatidis Radix (RIAF) had strong anti-inflammatory activity, but the mechanism of action was not well elucidated. Lipopolysaccharide- (LPS-) induced RAW264.7 cells were employed to observe the anti-inflammatory activity of RIAF. The level of interleukin-1*β* (IL-1*β*), tumor necrosis factor-*α* (TNF-*α*), nitric oxide (NO), prostaglandin E_2_ (PGE_2_), and interleukin-6 (IL-6) was determined by enzyme-linked immunosorbent assay kits. Western blot was performed to quantify the expression of extracellular signal-regulated kinase (ERK) 1/2, c-jun NH2-termianl kinase (JNK), p38, inducible NO synthetase (iNOS), cyclooxygenase (COX)-2, andnuclear factor-*κ*B (NF-*κ*B). Immunofluorescence assay and electrophoretic mobility shift assay (EMSA) were used to quantify the translocation and the binding-DNA activity of NF-*κ*B. RIAF could inhibit the secretion of inflammatory cytokines (PGE_2_, IL-6, IL-1*β*, and NO, other than TNF-*α*) in a dose-dependent manner. Further investigation showed that the expression of iNOS and COX-2 induced by LPS were downregulated by treatment with RIAF. Meanwhile, data from the signal pathway exhibited that RIAF significantly suppressed the phosphorylation of ERK1/2, JNK, and p38 and reduced the translocation of NF-*κ*B from the cytoplasm to nucleus, as well as the binding-DNA activity. The anti-inflammatory mechanism of action of RIAF was to reduce inflammation-associated gene expression (iNOS, COX-2, IL-1*β*, IL-6) by regulating the phosphorylation of the mitogen-activated protein kinases (MAPK) pathway and interventing the activation of the NF-*κ*B pathway, which partly illustrated the basis of treatment of Isatidis Radix on cold, fever, sore throat, mumps, and tonsillitis in clinics.

## 1. Introduction

Isatidis Radix, the root of *Isatis indigotica* Fort, has been used to treat colds, fever, sore throat, mumps, and tonsillitis in clinics for thousands of years in China [1, 2]. Inflammatory response is a common process of the diseases associated with bacterial or viral infections on the upper respiratory tract [3–5]. Isatidis Radix was also reported to have the anti-inﬂammatory [1–3], antivirus [6], antibacterial [7], and immune regulatory effects [8], which were attributed to syringic acid, isoquinoline derivatives, and flavonoids [8–10]. In a previous study, most research efforts focused on screening the active components against inflammation, and the acidic fraction of Isatidis Radix (RIAF) showed more strong anti-inflammatory activity than the neutral/basic fraction in lipopolysaccharide- (LPS-) induced RAW264.7 cells. However, the molecular mechanism of RIAF was unclear.

When colds, sore throat, and mumps occur, resident macrophages are activated. Some early cytokines such as interleukin-1*β* (IL-1*β*) and tumor necrosis factor-*α* (TNF-*α*) are upregulated by nuclear factor-*κ*B (NF-*κ*B) [11–13] and/or the mitogen-activated protein kinase (MAPK) signaling pathway [14], then adjacent cells are stimulated to release various chemokines, and neutrophils are recruited to arrange inflammatory response [15]. Meanwhile, cellular damage leads some key enzyme activation (such as cyclooxygenase-2/COX-2 and inducible NO synthetase/iNOS) to produce high levels of prostaglandins (PGE_2_) and nitric oxide (NO) in the airway epithelium [16]. PGE_2_ played a key role in the inflammation-related symptoms of common cold such as sore throat, myalgia, fever, nasal congestion, and rhinorrhoea [17, 18]. NO may contribute to inflammation by not only promoting eosinophil infiltration but also increasing microvascular plasma extravasation in the airways [19]. As a classic model of inflammation, the LPS-induced RAW264.7 macrophages cell was used to evaluate the anti-inflammatory activity and to illustrate the molecular mechanism of natural products or mixture. Therefore, it was employed to uncover the molecular mechanism of the acidic fraction of *Isatidis Radix* (RIAF) against inflammatory response.

## 2. Materials and Methods

### 2.1. Chemicals and Reagents

Isatidis Radix was purchased from Anhui Province in China and was identiﬁed by Professor Qi-Nan Wu, a botanist of Nanjing University of Chinese Medicine (Nanjing, China). A voucher specimen (NJUCM-20171106) was then deposited at a herbarium at the abovementioned location. RAW264.7 cell lines were provided by the Chinese Academy of Medical Sciences (Beijing, China). The enzyme-linked immunosorbent assay (ELISA) kits for TNF-*α*, IL-1*β* and interleukin-6 (IL-6) were obtained from eBioscience (Vienna, Austria). Prostaglandin E_2_ (PGE_2_) ELISA kits were purchased from Enzo Life Science (Farmingdale, NY, USA). Antibodies against inducible NO synthetase (iNOS), cyclooxygenase-2 (COX-2), phosphorylated or total p65, extracellular signal-regulated kinase (ERK) 1/2-Thr202/Tyr204, c-jun NH2-termianl kinase (JNK)-Thr183/Tyr185, and p38-Thr180/Tyr182 were obtained from Cell Signaling Technology (Beverly, MA, USA). DyLight 488-conjugated secondary antibody, horse radish peroxidase-conjugated secondary antibodies, and primary antibody against *β*-actin were purchased from Santa Cruz Biotechnology (Santa Cruz, CA, USA). Dulbecco's modified Eagle's medium (DMEM) and fetal bovine serum (FBS) were produced by Kaiji Biotechnology (Nanjing, China) and Sijiqing (Hangzhou, China), respectively. LPS (*Escherichia coli* O55:B5), 3-(4, 5-dimethylthiazol-2-yl)-2, 5-diphenyltetrazoliumbromide (MTT), 4′, 6-diamidino-2-phenylindole (DAPI), and cell-culture-grade DMSO were from Sigma-Aldrich (St. Louis, MO, USA).

### 2.2. Preparation of RIAF

Dried Isatidis Radix (4.0 kg) was decocted twice with distilled water (1 : 10 and then 1 : 8, w/v) for 1 h, and the total filtrates were concentrated to approximately 8 L in a vacuum evaporator at 60°C. 95% ethanol (v/v) was added in the concentrated extract up to 60% (v/v) and kept in a low-temperature bath for more than 12 h. After that, the supernatant was collected by a round of centrifugation at 3000 × g for 30 min and evaporated under vacuum to approximately 3.5 L. The solution was acidified to pH 5 with HCl and loaded onto a column packed with cation exchange resins (001 × 7, Cangzhou Bon Adsorber Technology Co., Ltd., Cangzhou, China), and the column was washed with water. The eluents were collected (35 L), alkalized to pH 9 with NaOH, and loaded onto another column packed with anion exchange resins (201 × 7, Cangzhou Bon Adsorber Technology Co., Ltd., Cangzhou, China). After the column was washed with water and 70% ethanol (pH 2), respectively, the ethanol eluents were evaporated under vacuum. It was subjected to D101 macroporous resin and eluted with a gradient ethanol-water (0, 30%, 50%, 95%). The combined ethanol eluents were dried by using a vacuum-drier at 60°C. The yield was 0.64%, and the purity was 89.52% by acid-base titration with phenolphthalein as the indicator, so the fraction was considered as the acidic fraction of Isatidis Radix (RIAF). The chemical characterization of RIAF was performed by HPLC according to the previous study [7], and several compounds such as syringic acid, salicylie acid, benzoic acid, caffeic acid, and 2-amino benzoic acid were identified.

### 2.3. Cell Culture and Sample Preparation

RAW264.7 cell lines were cultured in DMEM containing 10% FBS, 100 U/mL penicillin, and 100 *μ*g/mL streptomycin at 37°C with humidified atmosphere containing 5% CO_2._ The stock solution (5 mg/mL) of RIAF was prepared in deionized water.

### 2.4. Cell Viability Assay

RAW264.7 cells (1 × 10^4^ cells/well) were seeded in a 96-well plate overnight and pretreated by RIAF for 24 h at various concentrations (50, 100, 250, and 500 *μ*g/mL). After MTT (5 mg/mL), 20 *μ*L was added in each well, and cells were incubated for another 4 h. The supernatant was removed, and 150 *μ*L DMSO was added in each well. The optical absorbance at 490 nm was measured using a microplate reader (Molecular Devices, Menlo Park, USA).

### 2.5. Measurement of Cytokine Levels

RAW264.7 cells were plated in a 24-well plate at a density of 5 × 10^4^ cells/well for PGE_2_ and TNF-*α*, as well as 2 × 10^5^ cells/well and 1 × 10^5^ cells/well for IL-1*β* and IL-6. After 24 h, RAW264.7 cells were preincubated with RIAF (125, 250, and 500 *μ*g/mL) for 1 h and then stimulated by LPS (1 *μ*g/mL) for 24 h. The levels of PGE_2_, TNF-*α*, IL-1*β*, and IL-6 in supernatants were quantified by ELISA kits according to the manufacturer's instruction.

### 2.6. Measurement of Nitric Oxide (NO)

RAW264.7 cells (2 × 10^5^ cells/well) were seeded in 96-well plates overnight. Subsequently, RAW264.7 cells were treated with RIAF (125, 250, and 500 *μ*g/mL) for 24 h at the presence or absence of LPS (1 *μ*g/mL). NO level in the supernatant was detected by the Griess reaction [20]. Briefly, 100 *μ*L of the Griess reagent (1% sulfanilamide and 0.1% naphthylenediamine in 2.5% phosphoric acid) was mixed with an equal volume of the supernatant. The optical densities were measured at 540 nm after incubation in dark for 10 min.

### 2.7. Western Blot Analysis

RAW264.7 cells were pretreated with RIAF (125, 250 and 500 *μ*g/mL) for 1 h and stimulated with LPS (1 *μ*g/mL) for 20 min. Protein samples were prepared through lysing cells in lysis buffer for 10 min on ice. The total level of proteins was determined by bicinchoninic acid (BCA) assay (Sigma Aldrich, St. Louis, MO, USA). After being separated by 10% SDS-PAGE, the proteins of the sample were moved from within gel onto polyvinylidene difluoride (PVDF) membranes (Millipore, Billerica, MA, USA). After being blocked with 5% nonfat dry milk in PBS buffer for 1 h at room temperature, PVDF membranes were washed five times in PBS containing 0.1% Tween 20 (PBST) for 5 min. The blots were incubated with specific primary antibodies in PBST containing 3% bovine serum albumin (BSA) at 4°C overnight and were, in turn, incubated with corresponding horseradish peroxidase-conjugated secondary antibodies for 1 h at room temperature. The bands were exposed to films with enhanced chemiluminescence detection reagents (Pierce, Rockford, IL, USA).

### 2.8. Nuclear Factor-*κ*B (NF-*κ*B) Nuclear Translocation Assay

RAW264.7 cells (1 × 10^4^ cells/well) were cultured in 96-well plates and pretreated with RIAF (125, 250 and 500 *μ*g/mL) for 1 h prior to incubation with LPS for 20 min. Cells were then fixed with a 4% paraformaldehyde for 20 min at 4°C, permeabilized with 0.3% Triton X-100 for 20 min, and blocked with 5% BSA for 1 h at room temperature. Subsequently, cells were incubated with primary anti-p-p65 antibody for 2 h at room temperature, followed by DyLight 488-conjugated secondary antibody. After being washed with PBS, cells were incubated in DAPI solution (10 *μ*g/mL) for 10 min in the dark. Images of the live cells were visualized by using a confocal laser scanning microscope (BIO-RAD, Hemel Hempstead, UK).

### 2.9. Electrophoretic Mobility Shift Assay (EMSA)

RAW264.7 cells were cultured in 100 mm dishes (2 × 10^6^ cells/dish) and grown until confluent. Cells were preincubated with RIAF (125, 250, and 500 *μ*g/mL) for 1 h and stimulated with LPS for 30 min. After being washed with PBS, cells were collected, and nuclear and cytosolic extracts were then prepared according to methods described by Wang et al. [21]. EMSA assay was performed as previously described [22]. In brief, oligonucleotide (5′-AGTTGAGGGGACTTTCCCAGGC-3′, IDTDNA Technologies; Coralville, IA) was synthesized, which was labeled with biotin for the gel retardation assay. Equal amounts of nuclear extracts (10 *μ*g) were incubated with oligonucleotide for 20 min in 10 *μ*L buffer (0.1 M Tris, pH 7.5, 0.5 M KCl, 10 mM dithiothreitol/DTT). The NF-*κ*B-DNA mixture was separated by a 5% nondenaturing polyacrylamide gel and then transferred to nylon membranes. The biotinylated DNA was detected with a LightShift chemiluminescent EMSA kit (Thermo scientific, Rockford, IL, USA).

### 2.10. Statistical Analysis

Results were expressed as the mean ± SD. Differences were analyzed by one-way analysis of variance (ANOVA) and Student's *t*-test. *p* < 0.05 was considered as significant.

## 3. Results

### 3.1. Effect of RIAF on the Viability of RAW264.7 Cells

MTT assay showed that RIAF had no cytotoxicity on RAW 264.7 cells at a range of 50∼500 *μ*g/mL (Table 1). Therefore, the test concentration was no more than 500 *μ*g/mL in following experiments.

### 3.2. Anti-Inflammatory Activity of RIAF on RAW264.7 Cells

**Figure 1:**
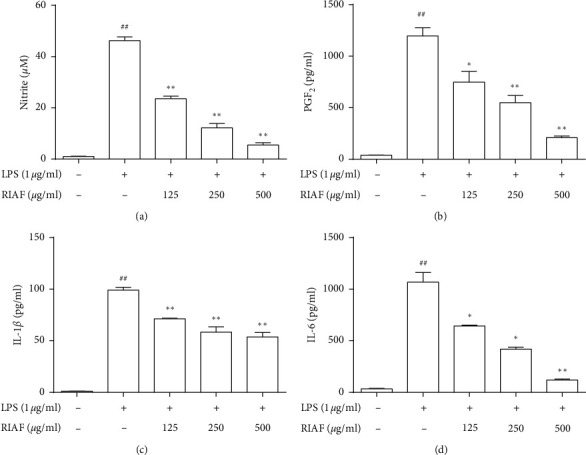
Effects of RIAF on inflammatory cytokines in LPS-induced RAW264.7 cells.

After RAW264.7 cells were stimulated by LPS, the levels of cytokines dramatically increased the production of NO, PGE_2_, IL-1*β*, IL-6, and TNF-*α* in the supernatants. Most cytokines were significantly reduced by pretreating with RIAF in a dose-dependent manner (as shown in Figure 1), of which the inhibitory rate was 90.04% (Figure 1(a)) and 91.28% (Figure 1(d)) for NO and IL-6 at high concentration. However, it was also noted that RIAF displayed a slight decline in the production of TNF-*α* (data not showed). The results suggested that RIAF had a good anti-inflammatory activity.

After being treated with RIAF at 125, 250, and 500 *μ*g/mL for 1 h, RAW264.7 cells were induced by LPS (1 *μ*g/mL) for 24 h, and the supernatant was collected for cytokine measurement with an ELISA kit. Data were expressed as means ± SD from three independent experiments. ^##^*p* < 0.01, compared with control. ^*∗*^*p* < 0.05 and ^*∗∗*^*p* < 0.01, compared with the model.

### 3.3. RIAF Inhibited LPS-Induced Expression of iNOS and COX-2

**Figure 2:**
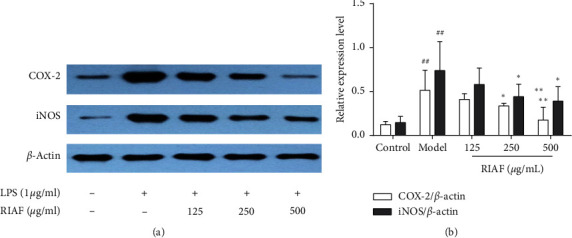
Effects of RIAF on the expression of COX-2 and iNOS in LPS-induced RAW264.7 cells.

Compared with the normal group, the expression of iNOS and COX-2 were significantly upregulated by LPS (1 *μ*g/mL) in RAW264.7 cells. The level of the two proteins was dramatically downregulated in a dose-dependent manner via pretreating with RIAF (Figure 2). COX-2 and iNOS were reported to play a vital role in the formation of NO and PGE_2_, respectively [20–23]. Thus, the results suggested that the activity of RIAF against NO and PGE_2_ was involved in iNOS and COX-2.

Cells were treated with RIAF at various concentrations for 1 h and induced by LPS (1 *μ*g/mL) for 20 min. Target proteins were separated by western blot, and the level was quantified by Image Tool 3.0. A representative of three independent experiments is shown in Figure 2. ^##^*p* < 0.01, compared with control. ^*∗*^*p* < 0.05 and ^*∗∗*^*p* < 0.01, compared with the model.

### 3.4. Effects of RIAF on LPS-Induced Phosphorylations of MAPKs

**Figure 3:**
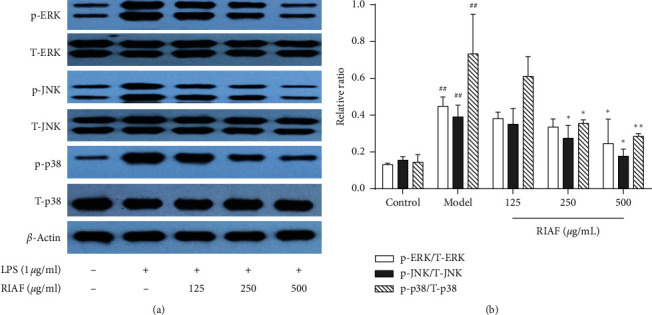
Effect of RIAF on MAPKs in LPS-induced RAW264.7 cells.

MAPKs are well recognized as an important signal pathway in LPS-induced macrophages activation, which is involved in the expression of specific gene association with inflammatory mediators such as IL-1*β*, IL-6, and PGE_2_ [14]. Meanwhile, Figure 3 also showed, significantly, the strong phosphorylation of MAPKs (ERK1/2-Thr202/Tyr204, JNK-Thr183/Tyr185, and p38-Thr180/Tyr182) in RAW264.7 cells after exposure to LPS for 20 min compared with the normal group. When RAW264.7 cells were pretreated by RIAF, the level of phosphorylation of the three proteins was obviously reduced in a concentration-dependent manner, and the amount of nonphosphorylated MAPKs was unaffected. The results indicated that RIAF could inhibit the inflammatory response through blocking the phosphorylation of extracellular ERK1/2, JNK, and p38 in LPS-induced RAW264.7 cells.

Cells were incubated with RIAF at various concentrations (125, 250, and 500 *μ*g/mL) for 1 h and then stimulated by LPS (1 *μ*g/mL) for 20 min. The levels of phosphorylated or total ERK1/2- Thr202/Tyr204, JNK-Thr183/Tyr185, and p38-Thr180/Tyr182 were determined by western blot, which were then quantified by Image Tool 3.0. A representative of three independent experiments is shown in Figure 3. p-ERK1/2, p-JNK, and p-p38: phosphorylation of ERK1/2-Thr202/Tyr204, JNK-Thr183/Tyr185, and p38-Thr180/Tyr182; T-ERK1/2, T-JNK, and T-p38: total ERK1/2, JNK, and p38. ^##^*p* < 0.01, compared with control. ^*∗*^*p* < 0.05 and ^*∗∗*^*p* < 0.01, compared with the model.

### 3.5. Effect of RIAF on Phosphorylation of NF-*κ*B in LPS-Induced RAW264.7 Cells

**Figure 4:**
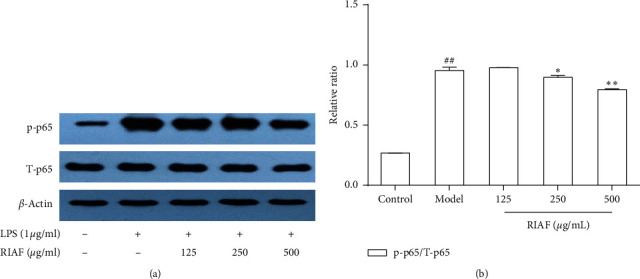
Effect of RIAF on phosphorylation of NF-*κ*B in LPS-induced RAW264.7 cells.

As another key regulator involving inflammatory response in macrophage cells [24], NF-*κ*B was also investigated to further reveal the anti-inflammatory mechanisms of RIAF. After RAW264.7 was stimulated by LPS, the phosphorylation of the NF-*κ*B p65 subunit was evidently increased. RIAF significantly inhibited the phosphorylation of p65-ser-536 in a dose-dependent manner (see Figure 4). This was why RIAF could reduce the production of IL-1*β* and IL-6 and the expression of iNOS and COX-2 in LPS-induced RAW264.7 cells.

Cells were incubated with RIAF at various concentrations (125, 250, and 500 *μ*g/mL) for 1 h and then stimulated by LPS (1 *μ*g/mL) for 20 min. The levels of p-p65, total p65, and *β*-actin were analyzed by western blot. p-p65: phosphorylations of p65; T-p65: total p65. The content of proteins was quantified as gray values by Image Tool 3.0. A representative of three independent experiments is shown in Figure 4. ^##^*p* < 0.01, compared with control. ^*∗*^*p* < 0.05 and ^*∗∗*^*p* < 0.01, compared with the model.

### 3.6. Effect of RIAF on Translocation of NF-*κ*B in LPS-Induced RAW264.7 Cells

**Figure 5:**
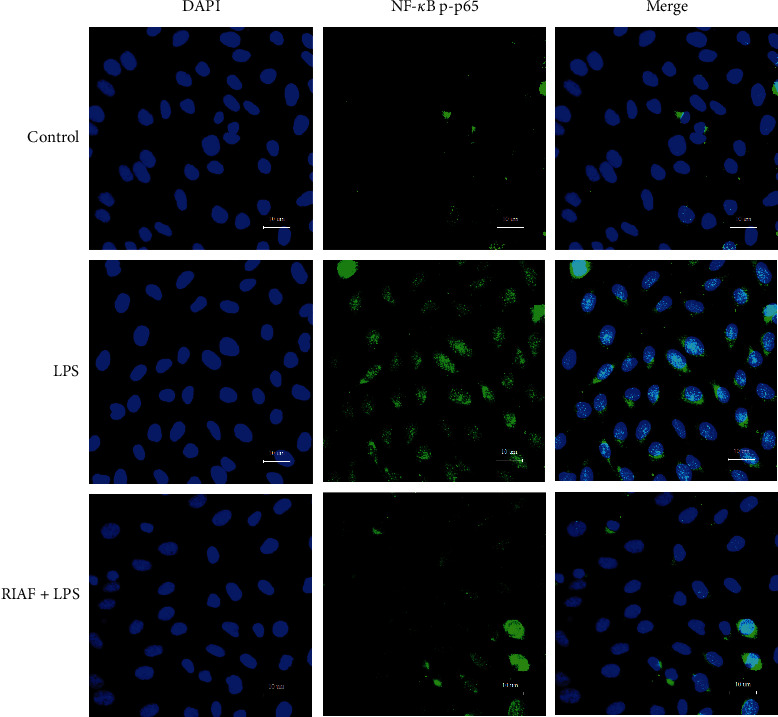
Effect of RIAF on the nuclear translocation of p-p65 protein.

NF-*κ*B nuclear translocation has been reported to be required for NF-*κ*B-dependent gene transcription following LPS stimulation. As shown in Figure 5, compared with the control group, p-p65 was clearly translocated into the nucleus from the cytoplasm of RAW264.7 cells with LPS. However, the level of the phosphorylated p65 in the nucleus was reduced by RIAF after pretreatment for 1 h followed by stimulation with LPS for 20 min, which indicated that RIAF could inhibit LPS-induced NF-*κ*B nuclear translocation in RAW264.7 cells under the concentration of 500 *μ*g/mL.

After being pretreated with RIAF for 1 h, cells were stimulated by LPS (1 *μ*g/mL) for 20 min. Nuclei were stained by DAPI (blue); p-p65 was labeled by DyLight 488-labeled immunostaining (green).

### 3.7. Effect of RIAF on LPS-Stimulated NF-*κ*B-DNA Binding Activity

**Figure 6:**
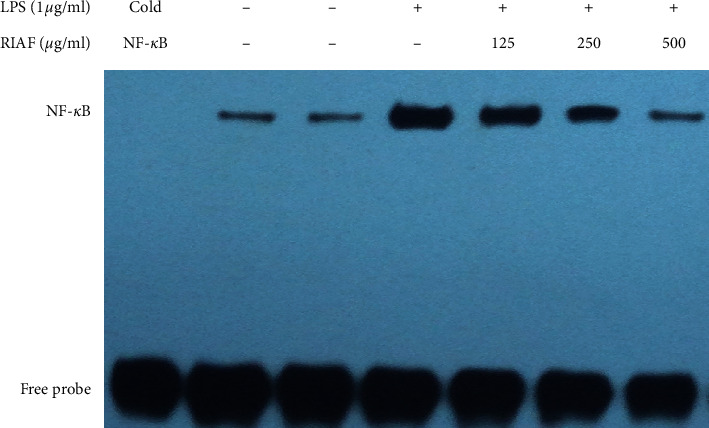
Effect of RIAF on LPS-stimulated NF-*κ*B-DNA complex formation in RAW264.7 cells.

The NF-*κ*B pathway plays a crucial role in mediating the transcription of a number of genes involved in inflammation on LPS-induced macrophage activation. NF-*κ*B-DNA binding is a directly initial trigger [25].  Figure 6 shows that LPS (1 *μ*g/mL, 30 min) treatment caused an evident increase in the DNA binding activity of NF-*κ*B, while NF-*κ*B DNA binding complexes were concentration-dependently reduced in RAW264.7 cells pretreated with RIAF.

After being pretreated with RIAF at various concentrations, RAW264.7 cells were exposed to LPS (1 *μ*g/mL) for another 20 min. Nuclear extracts were prepared through a Pierce nuclear extraction kit and separated by a 5% polyacrylamide gel with a biotin-labeled oligonucleotide containing the NF-*κ*B consensus sequence. Binding competition assays were run with a 100-fold amount of unlabeled NF-*κ*B oligonucleotide as the competitor (cold NF-*κ*B).  Figure 6 is a representative of three independent experiments.

## 4. Discussion

At present, nonsteroidal anti-inflammatory drugs ((NSAIDs) such as ibuprofen and celecoxib) are used to alleviate common symptoms associated with airway infection or cold (fever, sore throat, and mumps) [18]. Nevertheless, more and more debates have been increasingly reported over the appropriate use of those medications with the serious side effects of NSAIDs [26–28]. Many exterior-releasing and heat-clearing herbs were traditionally used to treat acute respiratory tract infection or complication in practice and showed a good anti-inflammatory activity. For example, Isatidis Radix, a heat-clearing and detoxicating herb, was reported to treat cold, fever, sore throat, and so on and had no obvious side effects [1–3], of which some compounds (epigoitrin, clemastanin B, indigoticoside A, syringic acid, caffeic acid, and salicylic acid) were also reported to inhibit the production of NO and PGE_2_. Most of the compounds had weak acid property due to the phenolic hydroxyl group [2]. It was consistent with the result that RIAF could inhibit inflammatory response.

Inflammation is a defensive response to foreign harmful stimuli, which is initiated by pathogen-associated molecular patterns (PAMPs) of microbial pathogens (such as LPS) in the process of cold, fever, sore throat, and mumps [29] and led to overproduction of cytokines such as NO, PGE_2_, TNF-*α*, IL-1*β*, and IL-6. MAPKs and NF-*κ*B were well known as two key signaling pathways involved in the abovementioned process [30, 31]. Among the cytokines, PGE_2_ was involved in sore throat via stimulating sensory nerves of pharynx and nasopharynx and increasing capillary permeability [15]. TNF-*α*, IL-1*β*, IL-6, and PGE_2_ were well regarded as endogenous pyrogens [17, 32]. *In vitro* assay confirmed that RIAF could significantly inhibit the abovementioned cytokines except for TNF-*α*, which implied that the therapeutic effect of RIAF was contributed to the anti-inflammatory activity.

iNOS and COX-2 were two rate-limiting enzymes in the NO or PGE_2_ synthesis pathway, and the expression was regulated by the MAPKs and NF-*κ*B pathway in LPS-induced inflammatory responses [23, 33, 34]. Western blot showed that RIAF could downregulate the expression of the two proteins, which indicated that the mechanism of RIAF against NO and PGE2 could be partially attributed to reducing the level of the two inducible enzymes on attack by foreign stimulants.

After the macrophage was stimulated by LPS, LPS could induce the phosphorylation of MAPKs or NF-*κ*B through toll-like receptor 4 and initiate the expression of cytokines (such as IL-1, IL-6, PGE_2_, and TNF) [35–37]. Among MAPKs, ERK1/2, JNK, and p38 had a central role in the regulation of cytokines [38]. p50-p65 dimers, an NF-*κ*B subgroup, played an important role in regulating cytokine production by residues the phosphorylation of the Ser^536^ residue on p65 and degradation of inhibitor kappa B (I*κ*B) [39]. The results revealed that RIAF could significantly suppress the phosphorylation of the MAPK and NF-*κ*B pathway. Nuclear translocation assay and EMSA further confirmed that RIAF could decrease the translocation of NF-*κ*B from the cytoplasm to nucleus and block binding of p65 to target genes. Therefore, it was reasonable that RIAF could inhibit inflammatory cytokines in LPS-induced RAW264.6 cells through regulating the phosphorylation of the MAPKs pathway and intervening the activation of the NF-*κ*B pathway. Meanwhile, a previous study [3] also showed that methanolic extracts of Isatidis Radix could significantly inhibit the release of inflammatory mediators (NO and PGE_2_) and proinflammatory cytokines (IL-6 and TNF-*α*) in LPS-stimulated RAW264.7 macrophages through inhibition of NF-*κ*B signaling. These results indicated that RIAF was the functional fraction to regulate the anti-inflammatory effect of Isatidis Radix. Yet, it should also note that RIAF was not able to inhibit TNF-*α*.

The reason for the selective inhibition of RIAF on NO, PGE2, IL-1*β*, and IL-6, but not TNF-*α*, was still unclear. Traditional Chinese medicine (TCM) is characterized by multiple components, weak effects, and synergistic effects. The overall effect of Isatidis Radix on the inflammatory response was not the result of the action of a single or several compounds, but the interaction of multiple compounds. Compared with Isatidis Radix, RIAF had some differences in the ratio and kind of the active compounds, which may influence the effect of RIAF on TNF-*α*. Indeed, further investigation is required to confirm this assumption.

**Figure 7:**
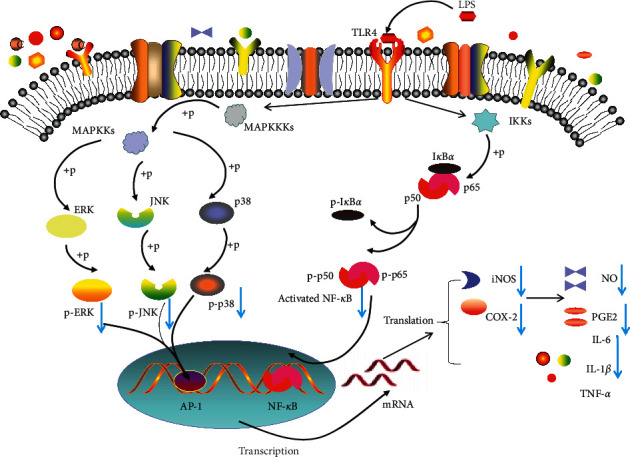
Putative RIAF inhibition pathway in macrophage-mediated inﬂammatory signaling.

In conclusion, our observations suggested that RIAF displayed a strong anti-inflammatory activity by suppressing MAPKs and NF-*κ*B activation to reduce inflammation-associated gene expression (iNOS, COX-2, IL-1*β*, and IL-6) (Figure 7). This implied that RIAF, at least in part, was the basis of the therapeutic efficacy of Isatidis Radix in cold, fever, sore throat, mumps, and tonsillitis. However, it is necessary to further investigate the activity and mechanism of RIAF in vivo. Meanwhile, compounds of RIAF against inflammatory responses should be identified in the future.

## Figures and Tables

**Table 1:** Effect of RIAF on the viability of RAW264.7 cells.

Concentration (*μ*g/mL)	Cell viability (%)
—	100 ± 0.00
500	103.97 ± 3.71
250	110.18 ± 5.20
100	113.59 ± 3.14
50	112.22 ± 1.08

## Data Availability

The underlying data supporting the results in the manuscirpt are included within the article.
